# A summary review of the development of using a brief imagery-competing task intervention (ICTI) for reducing intrusive memories of psychological trauma: applications in healthcare settings for both staff and patients

**DOI:** 10.1007/s44192-025-00205-6

**Published:** 2025-05-27

**Authors:** Julie Highfield, Lalitha Iyadurai, Emily A. Holmes

**Affiliations:** 1 Intensive Care Society, Breams Buildings, London, UK; 2 P1vital Products Ltd, Wallingford, Oxfordshire UK; 3 Department of Women’s and Children’s Health, Uppsala University, Akademiska sjukhuset, 751 85 Uppsala, Sweden

## Abstract

Psychological trauma for those utilising and delivering healthcare is common, and in particular the experience of repeated *and unwanted* intrusive memories (IM) of the trauma can occur. There are several psychological interventions that have been shown to be effective with the full syndrome of Post Traumatic Stress Disorder (PTSD), but researchers have only recently explored targeted interventions for IMs. This review provides a summary of a body of work on a behavioural technique called “Imagery Competing Task Intervention” (ICTI) for intrusive memories after trauma by Holmes and colleagues. The papers presented outline the underlying cognitive science, the historical development of the intervention, and its application to various different populations in healthcare settings including clinical tests of efficacy. Settings and populations include traumatic events experienced by emergency department patients and emergency caesarean section patients, as well as after work-related trauma experienced by intensive care staff and wider healthcare staff. Timing of ICTI intervention delivery has included the same day of trauma, within 72 h and for older memories weeks, months (or years) post-trauma. The intervention has been delivered with a guided session, which in some studies is in person and some remotely via digital health application. There is a brief overview of other related interventions. The ICTI approach shows potential scalability in trauma laden environments such as healthcare, where exposure is unlikely to be limited or managed and symptoms such as subclinical IMs are common. As such the intervention could be used in a preventing-and-treating approach and in subclinical-to-clinical samples who have IMs after exposure to psychological trauma. Future research would be needed to test ICTI as an intervention for the full syndrome of PTSD.

## Introduction

Psychological trauma is common and has increased for both hospital patients and healthcare staff since the COVID-19 pandemic [1, 2]. Based on a survey study of 24 nations, the global prevalence of exposure to one or more traumatic experiences in the general population^1^ is estimated to be 70.4% over the life course, with the majority having multiple traumatic exposure [3]. In terms of diagnostic criteria, psychological trauma is defined as exposure to “actual or threatened death, serious injury, or sexual violence” ([4] p. 271). When people experience psychologically traumatic events what can follow is an array of sensory memories which recurrently spring to mind bringing back the event together with strong emotions and impacting on the person’s functioning. Indeed, intrusive memories (IMs) are commonly experienced following traumatic events, especially initially. IMs are emotional, sensory, and primarily visual memories (mental imagery) of a traumatic event that intrude unwantedly and repeatedly into mind. They typically appear as brief scenes of ‘hotspot’ moments [5] within the wider event. They are a core clinical feature of both acute stress disorder [6] and longer term post-traumatic stress disorder (PTSD) [4]. An example from healthcare staff of a work-related trauma memory is having a sudden vivid mental image of a relative’s face at the bedside of a dying patient [7].

For some individuals, re-experiencing symptoms such as IMs, if experienced in the first few days following trauma, may abate over the next days and weeks, for others they have been associated with a diagnosis of PTSD at 1 year [8]. “Recurrent thoughts of the trauma”, encapsulating IMs, was repeatedly found to be one of the top PTSD symptoms that were centrally linked to other symptoms in a systematic review of network analysis studies of PTSD symptoms [9]. IMs also occur in other psychological disorders such as depression [10] and complex grief [11]. As such IMs can be considered both transdiagnostic and predictive. Therefore, to address such IMs easily accessible early interventions are required. Current evidence-based interventions such as trauma focussed cognitive behavioural therapy (CBT) or eye movement desensitisation and reprocessing (EMDR) mainly target the full syndrome of PTSD [12] and while highly important and valued treatments, they have restricted accessibility as they are delivered in psychotherapy settings. Interestingly, the updated NICE guidelines for PTSD now consider alternative interventions targeted at specific symptoms (rather than the full syndrome) in some circumstances, such as when other interventions are not available and in line with patient choice [12]. As mentioned, existing approaches are effective and important, and we note that ICTI is not conceived of as a replacement for existing evidence-based treatment, but rather a complementary approach that could be useful in a variety of circumstances such as in limited resource settings.

Frontline healthcare settings globally, such as hospital care and emergency care, are an example of where simple, accessible, and brief intervention approaches for staff and patients experiencing IMs after exposure to trauma would be beneficial. This is because access to existing evidence-based psychological interventions in more traditional outpatient mental health settings e.g. a psychiatry clinic or outpatient psychotherapy is challenging for those in frontline healthcare settings. Challenges for healthcare staff in particular include limited time windows for intervention, difficulty committing to a regular time slot and location, limited access to private spaces, and stigma. Further, the existing evidence-based psychological interventions have typically been developed for delivery *after* exposure to trauma has ended (e.g. after war combat, rape, or a motor vehicle accident). In contrast, for healthcare staff and patients in frontline healthcare settings, there is a possibility of ongoing and repeated experience of traumatic events. This means that feasible interventions must be brief, flexible in terms of timing and location, low in stigma and repeatable for multiple and ongoing trauma exposure. Scalability is also important given the extent of trauma worldwide beyond healthcare staff, for example for other first responders (e.g. police, fire service, humanitarian aid workers), after interpersonal violence, sexual assault, trauma-exposed people in war situations and/or those who are refugees [3]. There was a call in 2018 for the mental health science community to develop mechanistically driven psychological interventions that are more scalable [13].

Here we focus on the development of a new intervention approach targeting preventive and treatment efforts on the focal symptom of IMs after traumatic events, by Holmes and colleagues [14–16]. It is derived from mental health science [17]) and targets IMs via their perceptual, mental imagery-based nature using a so called “imagery competing task intervention” (ICTI) [7, 18]. The new intervention to reduce IMs aims to be readily repeatable for different or new traumatic events as well as brief, flexible, accessible and low in stigma. This approach first developed in the laboratory [14, 19, 20] has been developed across several trauma populations and settings in small scale clinical studies and case series, including with people who are refugees [21], inpatients with PTSD [22], a patient with bipolar disorder [23], and women following childhood trauma [24–26]. The imagery-competing task intervention has now been successfully utilised in randomised controlled trials with various groups in healthcare/hospital settings, including intensive care staff [7, 18], frontline healthcare workers [27], mothers following emergency caesarean sections [28–31], and patients attending the emergency department [32, 33]. One recent study with PTSD patients using a cross over design however did not replicate positive intervention effects [34]. An ICTI type approach has recently been proposed for use in a neonatal trauma setting [35]. Another study has combined a similar competing visuospatial task approach together with exposure-based treatment for PTSD as an adjunctive (rather than standalone) treatment in a clinical inpatient setting for PTSD [36]. In this review article, we focus on evidence from the randomised controlled trials that have tested ICTI as a standalone intervention in acute healthcare and hospital settings, with either staff or patients experiencing intrusive memories after traumatic events.

## Intrusive memories and trauma in healthcare populations

The very nature of frontline healthcare means that staff and patients will be exposed to distress, diseases, and death alongside joy, birth, and patient recovery. Potential exposure to psychological trauma is therefore inherent in frontline healthcare settings.

### Healthcare staff

Throughout the COVID-19 pandemic, frontline healthcare workers (such as those working in intensive care, emergency care, prehospital care, and acute wards) have been exposed to an increased number of potentially psychologically traumatic events, such as excess deaths of patients, and a stretching of resources alongside increasing demands for services, under conditions of duress due to the need for personal protective equipment (PPE). There is evidence to suggest frontline workers report high levels of IMs, even pre-pandemic: for example, 65% of emergency-room nurses reported IMs of work-related trauma, most commonly of failed resuscitations or events involving young people [37]. Meta-analysis estimates show that healthcare workers are twice as likely to develop PTSD compared to the general public [38]. During the COVID-19 pandemic, around 40% of a sample of intensive care staff in UK hospitals reported a level of symptoms consistent with a diagnosis of PTSD as of June/July 2020 [39]—five times higher than reported by a similar population in 2015 [40]. Although this work may be criticised for “eye of the storm” reporting, it certainly indicates the heightened distress and symptoms of psychological trauma healthcare workers experienced when the system was under extreme duress. Healthcare staff typically have high work demands, and work shifts, so our clinical experience suggests that they may not have the time to access the trauma-focused talking therapies such as trauma focussed cognitive behavioural therapy (CBT) or eye movement desensitisation and reprocessing (EMDR) recommended in evidence-based guidelines [12]. In addition, it is unlikely they have single incident trauma exposure, but have had repeated and possibly ongoing exposure to a number of traumatic events throughout their work, and during times such as the recent COVID-19 pandemic this exposure can surge.

### Patients

There are several areas of frontline healthcare that patients as well as staff may typically experience as psychologically traumatic, including physical trauma (i.e., serious injury to the body, such as broken bones, deep cuts, or concussion), intensive care, emergency care and emergency caesareans. Rates of PTSD can be high for these patients, and IMs are common. One study found that of 363 consecutive admissions to a level 1 physical trauma service 10% developed PTSD and major depressive disorder at 12 months [41]. In burn patients specifically, a meta-analysis found PTSD prevalence up to 45% in the first year [42]. Patients who have been through intensive care also have elevated rates of PTSD compared to trauma controls (17% vs 7%) [43]. One study found that 50% of acute respiratory distress patients who stayed in ICU had IMs [44]. An interview study found that the majority of patients had IMs of hallucinations/delusions from their time in intensive care, and the content of the IMs often merged real events (for example, involving staff, or medical procedures) with delusions and hallucinations [45]. A large survey of 957 patients attending a hospital emergency department following a motor vehicle accident found 23.1% PTSD prevalence at 3 months and 16.5% at 1 year [46].

Approximately a third (39%) of mothers develop postnatal posttraumatic stress disorder following emergency caesarean section (ECS) [47] compared to 5.6% following childbirth in general [48]. Examples of IMs following ECS include a mental image springing to mind of the screen of the foetal heart rate monitor indicating ‘Stop’ or seeing the face of the doctor announcing that the patient immediately needs an ECS. These IMs may be associated with problems with sleep, poor coping, and problems with breast feeding [49, 50]. Postnatal PTSD can impact the attachment relationship between mother and baby, increasing parental stress and impacting the baby’s development [17, 49]. Postnatal PTSD significantly contributes to the costs of perinatal mental health problems, estimated at £8.1 billion per year in the UK alone [51]. Overall, findings from across healthcare settings indicate that IMs from a variety of traumatic experiences related to health care are therefore not only problematic as symptoms in their own right, but also associated with costs to mental health and day-to-day functioning.

## Cognitive science which contributed to the development of ICTI

There are useful insights from cognitive science to allow us to understand the nature of IMs and how to potentially disrupt them. We know that IMs of trauma comprise sensory-perceptual mental images which have visuospatial components [5, 52]. It has been proposed that they occur due to excessive sensory processing during a psychologically traumatic event [53] which leads to sensory based, predominantly visual, memories of the trauma which spontaneously intrude into the mind. Memory consolidation theory suggests a potential time window of around six hours post trauma during which trauma memory is not yet stabilised and vulnerable to disruption [54, 55], although the exact time frame is debated. This early time frame post traumatic event (for example, on the same day of the trauma) presents a window of opportunity during which to disrupt the consolidation of imagery-based memory, for example -as we have suggested [14, 56] with a engaging in a task that competes with mental imagery.

As IMs are predominantly visual, one way to disrupt this form of imagery-based memory is with an imagery competing task, as the hypothesis is that such tasks compete for resources with the brain’s sensory-perceptual resources. Some experimental research suggests that those competing tasks that engage visuospatial processing, compared to say more verbal tasks, are likely to be the most successful in reducing the occurrence of subsequent intrusive visual memories of trauma [57] (it is also possible some tasks with general working memory taxing can also be beneficial [58]). Disrupting the visual aspects of trauma memory during its consolidation is predicted to render the memory less ‘overly’ perceptual, and thus less intrusive as it will be less easily triggered by sensory cues in the environment. Research suggests that after a trauma memory is brought to mind, when we aim to disrupt visual aspects of the traumatic memory by actively engaging in visuospatial tasks, it is possible to reduce the number of intrusive memories [59–61].

The cognitive behavioural formulation of trauma memory suggests that it is only the discrete points within the memory referred to as ‘hotspots’, that later become intrusive memories rather than the *entire* memory of the event [5, 62–64]). So, to reduce intrusive memories, the individual does not need to go through the memory of the whole trauma. Instead, we can take a more “precision” based approach and target these briefer hotspot moments selectively and thereby compete for resources with the consolidation of just these parts. This has the advantage that this is less distressing than talking about the trauma in more detail, which can be difficult for some individuals. Since people typically begin to re-experience intrusions even soon after the event [5], hotspots can be swiftly ascertained (even in the first hours after a trauma has occurred) by asking about “worst moments” within the wider trauma memory or specific memories that are already intrusive [33]. To summarise, for the ICTI intervention to work, we need to pinpoint precise moments within the trauma memory that are likely to recur as intrusive memories before doing the task. This is done via the use of a reminder cue to each of those moments.

What about time intervals longer than a few hours after trauma? A similar hypothesis applies to established memories of trauma (from a day or more after the trauma). Inspired by research on “memory reconsolidation” [65–67] suggesting it may be possible to update older, established memories, we further developed the intervention approach to reactivate older trauma memory hotspots using a brief reminder cue, making them once again vulnerable to disruption using a competing visuospatial cognitive task thought to compete with mental imagery [68] as supported by laboratory study findings in the first days after experimental trauma [20, 69].

In summary, actively engaging in an imagery competing task after a reminder cue to precise hotspot moments either in the immediate aftermath of a traumatic event (e.g. the first six hours post-trauma), or at a longer time after the traumatic event (i.e. for older trauma memories days, weeks or years later), is predicted to reduce the occurrence of subsequent intrusive memories of the trauma via competing with sensory aspects of the trauma memory before it has been fully (re)consolidated. Comparing the imagery competing task intervention as used on the same day as trauma with that for older memories, the similarity in procedures is the reminder cue and task, whilst the difference in the case for older memories is a longer time allowed between cue and task (so memories can become labile) of approximately 10 min to the task.

## The development of the imagery competing task intervention for IMs

Holmes and colleagues have been developing an imagery competing task intervention approach under controlled laboratory settings for a number of years, finding that a procedure including doing complex visuospatial tasks (e.g. complex concealed pattern tapping) during or soon after experimental trauma have led to a reduction in the number of subsequent IMs [14, 19, 70–72]. In contrast, more verbal tasks typically do not, with inconsistent findings whereby in some studies they may reduce intrusions in other studies they may even increase intrusions indicating potential for harm ([19, 70, 73, 74]; see [57]). Used within the wider ICTI intervention procedure, visuospatial tasks are more than mere distraction; they are thought to provide modality-specific interference with sensory (visuospatial) aspects of intrusive memory, as described above. The computer game ‘Tetris®’ [75] is a task that engages visuospatial processing [76] and has been tested as a visuospatial task component part of the brief imagery-competing intervention to reduce IMs. It is noted that merely playing Tetris alone is not predicted to reduce IMs, rather the game should be used as one component of the wider intervention procedure. Laboratory studies have found the intervention approach to be effective in reducing the number of IMs compared with no task and active control tasks [19, 20, 70].

The intervention has subsequently been translated for use in a number of clinical settings involving healthcare patients and staff. In these settings, this intervention approach offered a number of advantages, as it is brief (approximately 25 min per session), flexible (can be used in different locations and on different devices) and minimised stigma (involving a simple computer game rather than talking therapy approach). Here we describe several randomised controlled trials as a summary of the development of this approach-whereby two trials used the intervention on the same day as the traumatic event, one within 72 h of the trauma, and two with for much older memories of trauma.

A brief imagery competing task (including Tetris®) intervention was utilised by Horsch and colleagues with women following emergency caesarean section (ECS) [28]. Fifty-six women after ECS were randomized to one of two parallel groups in a 1:1 ratio: intervention of usual care plus the brief imagery-competing task or the control group of usual care. The intervention group were in a reminder context for their traumatic event (here, the hospital ward after surgery) and engaged in Tetris® gameplay for 15 min within 6 h following their ECS. The primary outcome was the number of intrusive traumatic memories related to the ECS recorded in a diary for the week post-ECS. Compared with controls, the intervention group reported 48% fewer intrusive traumatic memories over 1 week (mean per week 4.77 vs. 9.22) and there was a trend towards reduced acute stress re-experiencing symptoms after 1 week. 72% of women rated the intervention “rather” to “extremely” acceptable.

**Fig. 1:**
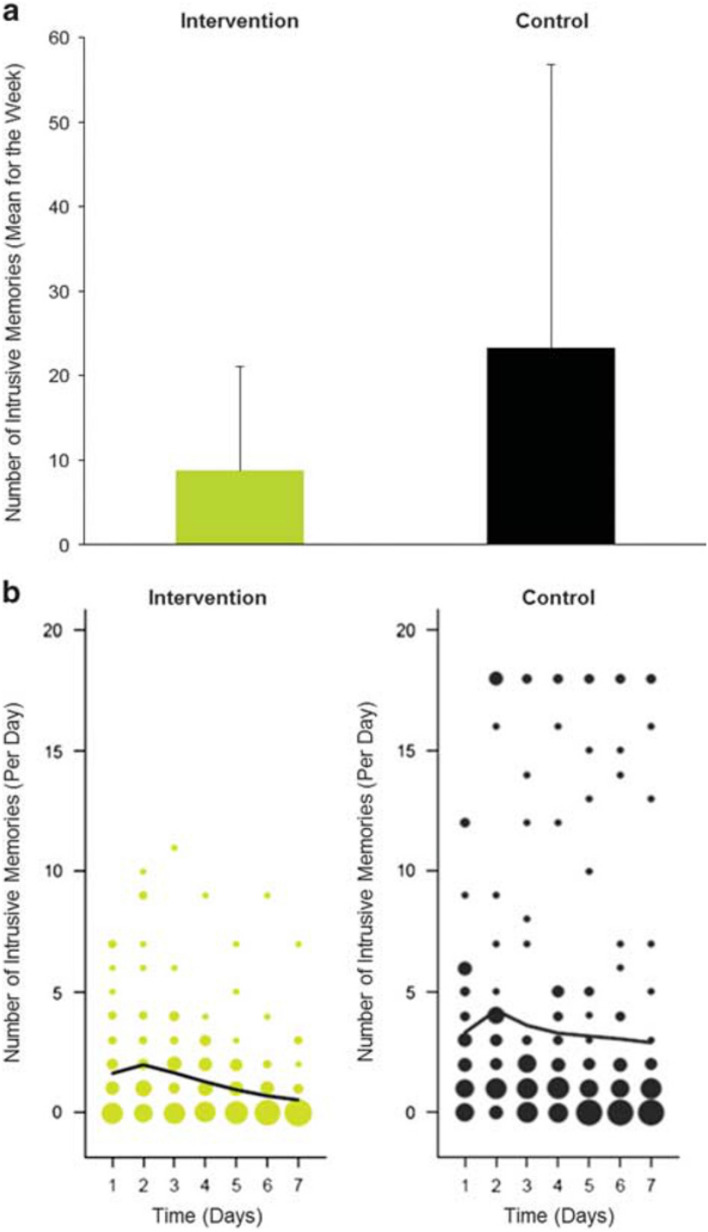
Number of intrusive memories of the traumatic event in the intervention and control conditions, figure and legend taken from Iyadurai et al. [33]. **a** Mean number of intrusive memories recorded in a daily diary during the week following a traumatic motor vehicle accident (intention-to-treat analysis). Intervention condition = cognitive task (trauma memory reminder cue plus Tetris computer game play); Control condition = written activity log. There was a significant difference between the intervention condition (n = 37, M = 8.73, s.d. = 11.55, range 0–55) and the control condition (n = 34, M = 23.26, s.d. = 32.99, range 0–120): t(69) = 2.80, *P* = 0.005, d = 0.67, 95% CI 0.18, 1.14. Error bars show standard deviations. **b** Frequency scatter graphs (exploratory analysis) showing the time course of the number of intrusive memories recorded in a diary from day 1 (day of trauma) to day 7 for participants who returned the diary in the intervention condition (n = 34) and control condition (n = 33). The size of the circles represents the number of participants who reported the indicated number of intrusive memories on that particular day, scaled separately for each condition. The solid lines are the fit of the generalized additive model to summarize the number of intrusive memories through the 7-day period

Iyadurai and colleagues tested the brief imagery competing task intervention (including Tetris®) in a randomised controlled trial with emergency department patients [33]. The one session intervention involved a trauma memory reminder cue (briefly telling the researcher the worst moments of the trauma) plus about 20 min of Tetris® game play (with mental rotation) versus an attention-placebo control of a written activity log for the same duration. Both intervention and control conditions were delivered in an emergency department within 6 h of a motor vehicle accident. The primary outcome was the number of intrusive trauma memories in the subsequent week. In the intervention condition there were 62% fewer intrusive memories overall than in the control condition (8.73 vs. 23.26), and intrusion incidence declined more quickly (see Fig. 1). Participants found the intervention easy, helpful, and minimally distressing. Feedback from a number of participants indicated that, whilst playing Tetris seemed strange at first, they enjoyed it and found it took their mind of what had happened whilst they were waiting in the emergency department.

The next step in this research followed with an investigation into the use of the intervention with patients presenting to the emergency department (ED) in a Swedish hospital [32]. Kanstrup and colleagues conducted an exploratory pilot randomised controlled trial of the imagery competing task intervention with 41 patients. Participants were randomly allocated within 72 h of presenting to the ED to either the intervention of a reminder memory cue and visuospatial task (Tetris with instructions to use mental rotation while playing the game), or the active control group of a podcast. The study differed from Iyadurai et al. [33] by including people with a broader range of trauma (e.g. physical assault as well as motor vehicle accidents) and extending the time frame for follow up to over a month. Compared to the control condition, participants in the intervention condition reported 48% fewer intrusive memories of trauma (3.85 vs. 7.37) at week 1 and 90% fewer (0.28 vs. 2.89) at week 5.

Next steps were then taken by the research team to develop this approach as an intervention for UK intensive care staff working in the COVID-19 pandemic who were already experiencing IMs outside the 6-h window, so this could be for days, weeks or months post traumatic event [7, 18]. Critically, these workers were also likely to be continuing to be exposed to ongoing work-based trauma. Of note, ongoing exposure to trauma appears to be an exclusion criterion for many clinical trials of interventions post-trauma, whereby the intervention is conducted after the trauma exposure has ended (e.g. after combat, a motor vehicle accident etc.) so there are few interventions available for those who face ongoing trauma exposure. In this next trial [7, 18] participants were included if they experienced at least one work related traumatic event and at least three IMs in the week prior to recruitment. This clinical trial extended previous trials by testing (a) treatment of established intrusive memories (rather than only prevention in the early aftermath of trauma) (b) an integrated remotely delivered digitised version of the intervention on the digital platform i-Spero®, (c) effect on other clinical symptoms and work functioning, (d) use for those exposed to repeated, ongoing trauma (rather than only trauma episodes that have ended) with possible repeat administrations during the study period, and (e) novel trial methods using an adaptive optimisation approach. A two-arm, parallel-group, randomised, trial was used, with an adaptive Bayesian optimisation phase (for discussion of the statistical approach, see Ramineni [18]). Participants were randomised to receive immediate or delayed (after 4 weeks) access to the intervention.

**Fig. 2:**
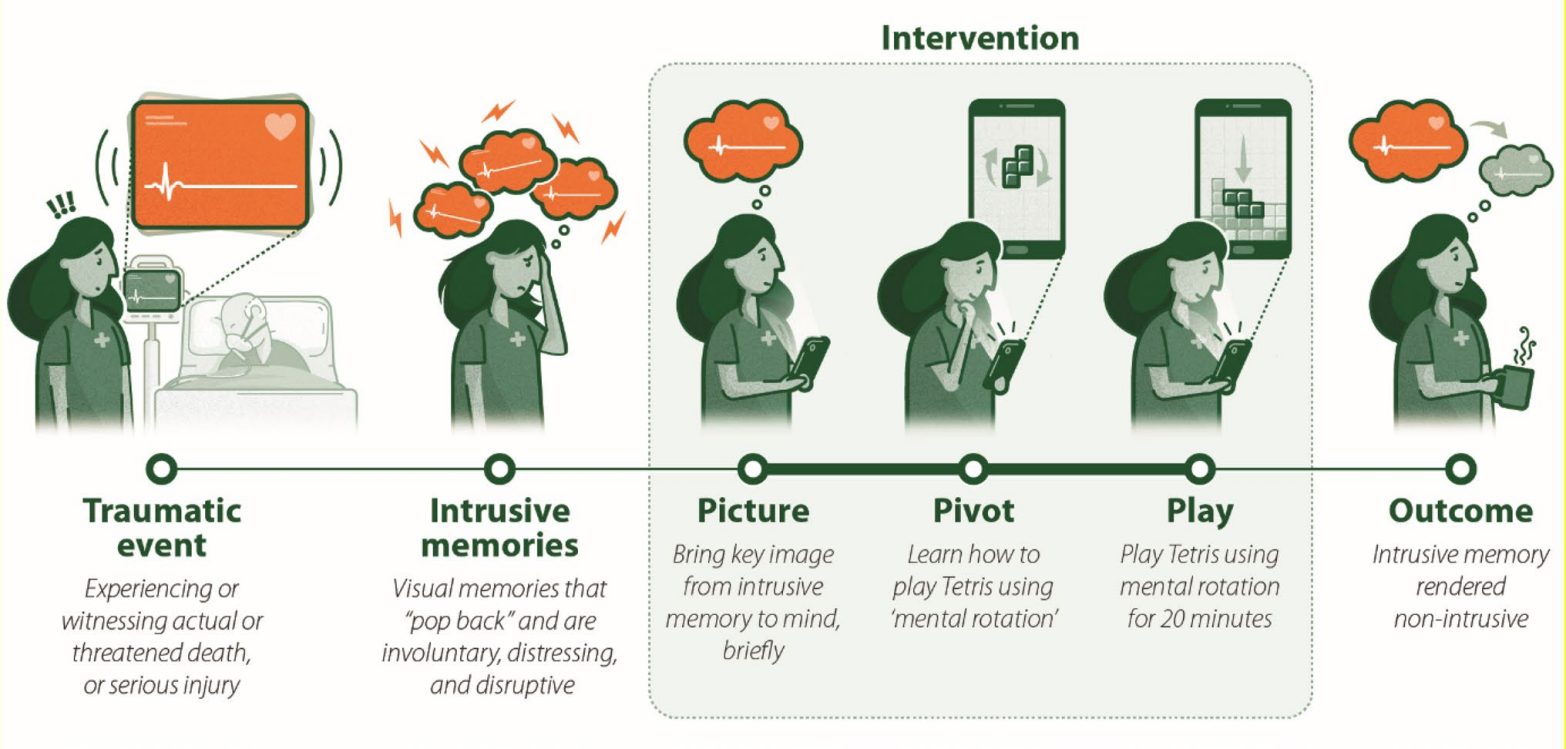
Graphic illustrating the key components of the imagery-competing task intervention. Illustration developed by © Diogo Guerra

The digitised version of the intervention was developed to allow the intervention to be administered remotely first by a guided session with the researcher, and then it could be self-administered, and overall be brief, simple and easy to use. Being accessible via the internet from a smartphone or computer the intervention can be completed as many times as needed when and where is most convenient for the user. A digitised version of the intervention can also be deployed more rapidly and at scale compared to those that required professional therapist contact. Participants recorded IMs during the baseline week before randomisation, then participants in the first arm had immediate access to the intervention, while those in the comparator arm monitored their symptoms for 4 weeks alongside usual care (defined as receiving any treatment they would otherwise access) and then had delayed access to the intervention. The imagery-competing task intervention consisted of a brief reminder cue to a specific intrusive memory (just a few words), followed by playing the computer game Tetris® for 20 min (which includes the extra time need for established memories of trauma) with instructions to use mental rotation while playing the game (see Fig. 2). The first session was guided by a researcher (remotely via video call to reduce infection risk during the pandemic) which included help for the participant to navigate the digital platform and thereafter the intervention was repeatable and self-guided. All components were integrated on a secure web platform (i-Spero®) accessed via smartphone, tablet, or computer. The intervention package also included step-by-step written guidance, instructional video animations, embedded ratings and integrated methods for recording and tracking the number of intrusive memories to guide intervention use.

Intrusive memories were recorded by participants in a brief daily online diary for 7 days at baseline, week 4, and week 8 (delayed arm). The primary outcome was the number of IMs of trauma during week 4, controlling for baseline week. Prior to final analysis, sequential Bayesian analyses were conducted to inform early stopping of the trial prior to the planned maximum recruitment allowing optimisation of recruitment, with 86 participants in total, equally across each arm.

**Fig. 3:**
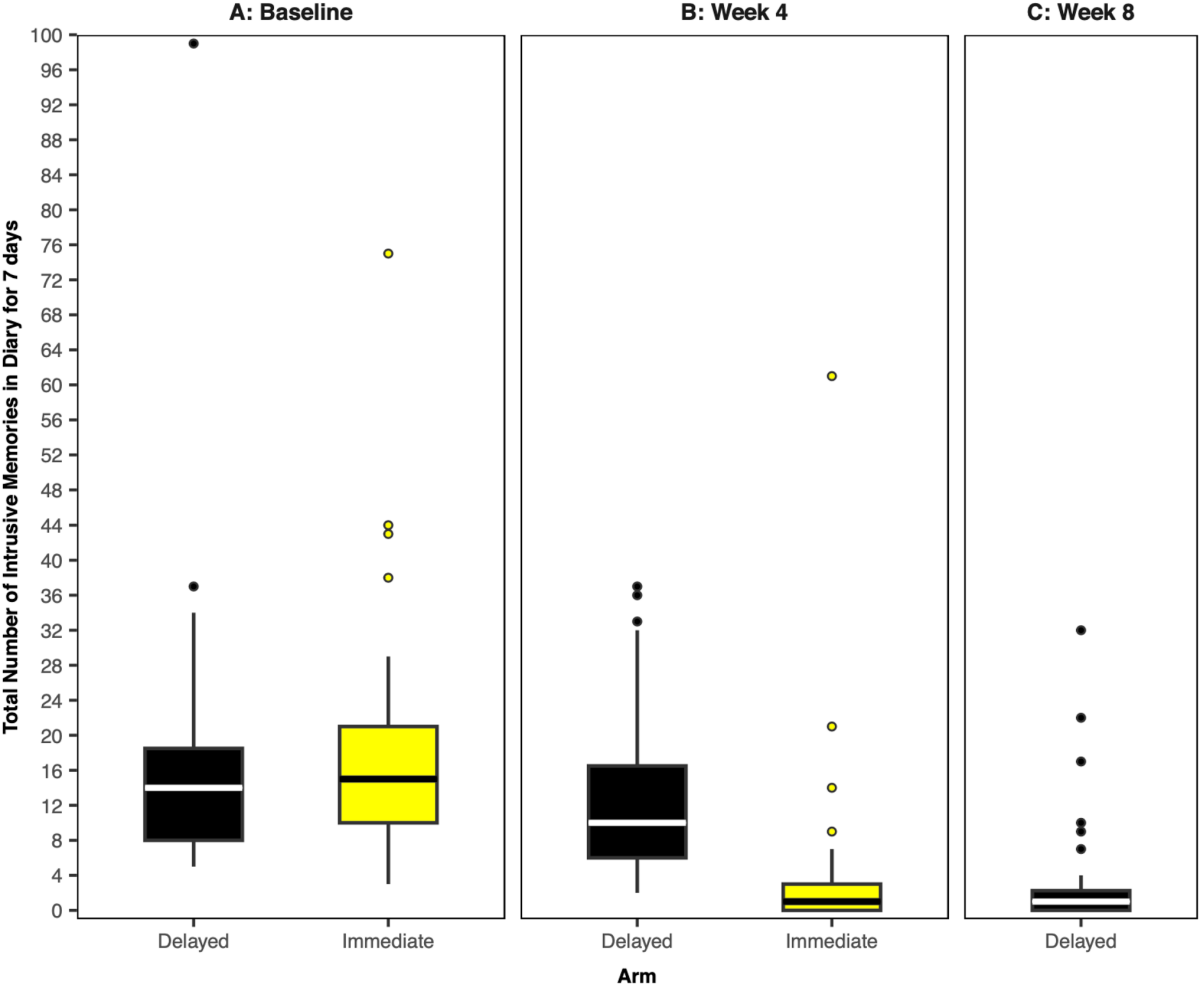
Figure and legend reproduced from Iyadurai et al. [7]. Boxplots showing number of intrusive memories of traumatic events. The midline of each boxplot is the median value, with the upper and lower limits of the box being the third and first quartile (75 th and 25 th percentile), and the whiskers covering 1.5 times the interquartile range (IQR). The dots depict outliers (each dot represents one participant that departed by more than 1.5 times the IQR above the third quartile and below the first quartile). All outliers are included in this figure. **A** Baseline measure for each arm. Number of intrusive memories of traumatic events recorded by participants in a brief daily online intrusive memory diary for 7 days during the baseline week for both arms (black = delayed arm; n = 39: usual care for 4 weeks; yellow = immediate arm; n = 37: immediate access to the intervention following the baseline week), showing that the two arms did not differ at baseline (i.e., before the intervention was provided to either arm). **B** Primary outcome measure for each arm. Number of intrusive memories of traumatic events recorded by participants in the daily online intrusive memory diary for 7 days during week 4 for each arm (black = delayed arm; n = 39: usual care for 4 weeks; yellow = immediate arm; n = 36: immediate access to the intervention following the baseline week). The intervention consisted of a cognitive task involving a trauma reminder-cue plus Tetris® computer gameplay using mental rotation plus symptom monitoring. The immediate access arm had fewer intrusive memories at week 4 compared to the delayed arm, and the number of intrusive memories for the immediate arm decreased between the baseline week and week 4. **C** Secondary outcome measure for the delayed intervention arm. Number of intrusive memories of traumatic events recorded by participants in a brief daily online intrusive memory diary for 7 days during week 8 for the delayed arm (black; n = 32: usual care for 4 weeks followed by access to the intervention for 4 weeks), showing that the number of intrusive memories decreased between week 4 and week 8

ICU staff reported high numbers of work-related traumatic events prior to starting the study (approx. 37 traumas per participant) and a high level of IMs (combined median = 14, IQR = 9–20)—neither differed between the immediate intervention and delayed comparator arms. Critically, after receiving the intervention, the immediate arm reported significantly fewer IMs than the delayed arm (median = 1, IQR = 0–13 vs. median = 10, IQR = 6–7), indicating a substantial drop in IMs from baseline (78%). After crossover (that is getting access to the intervention in the arm that had not had it before), the delayed arm also showed a significant reduction in IMs at week 8 compared to week 4 (73%), again to a median of 1 per week. Figure 3 below, taken from Iyadurai et al. [7], shows the total number of intrusive memories in the two arms at baseline, week 4, and week 8 (delayed arm only).

At week 4, the immediate intervention arm participants had significantly lower symptoms of PTSD, insomnia, anxiety and post-trauma distress, with no difference in depression, than the comparator delayed arm. This corresponding reduction in clinical symptoms of PTSD and anxiety suggests that targeting this single symptom of PTSD (IMs) may have downstream benefits for other associated mental health symptoms. This is consistent, for example, with network models of PTSD, which indicate that intrusions are centrally linked to other symptoms [77, 78]. Work engagement was significantly higher and burnout significantly lower in participants in the immediate intervention arm compared to those in the delayed arm at 4 weeks. There was no significant difference in number of sick days or intention to leave their job. Adverse events were surveyed throughout the trial by scheduled questions and free report. All adverse events were unrelated to the study. The intervention was found safe and acceptable to participants. Therefore, this remotely delivered, brief, flexible, low-intensity intervention offers one potential solution to help address the impact of work-related trauma (such as here during the pandemic) on the mental health and functioning of healthcare workers at least in terms of intrusive memories of work-related trauma, with potential for future scalability.

A similar pattern of results has been shown in a broader sample of healthcare workers in Sweden in the EKUT-P RCT) [79], also in a single guided session but which used another digital platform to deliver ICTI (this more basic platform required participants to “hop” between applications for gameplay, rather than being a integrated digital platform like i-Spero® used in the GAINS study). In the EKUT-P study, the intervention was compared to an active comparator (a podcast) also delivered via smartphone in a single guided session for a similar duration of time. Results showed that compared to active control, at 5 weeks after the single guided session of ICTI, there was a reduction in IMs by about approximately 75%. As well as showing a positive treatment effect on IMs, the intervention also had a beneficial effect on symptoms of PTSD more broadly—with this difference lasting during the follow up period. At the 6 months follow up, compared to active control, in the intervention group the score on a measure of PTSD symptoms was approximately half. Tests of replication are warranted and a further clinical trial is underway with an additional active control comparator with an alternative cognitive task which delivered using the same digital platform (i-Spero®) and a long term follow up over 6 months.

In each of the above five studies, the single guided session of the intervention was delivered by trained researchers, ranging from research assistants to qualified clinical psychologists. All researchers received training, feedback, and ongoing monitoring and supervision in use of the methods from clinical researchers who were experienced in delivering the intervention. Training is a critical part of guided intervention delivery, to promote treatment fidelity and protocol adherence.

## Alternative and potentially related intervention approaches

As mentioned, current evidence-based psychological treatments for PTSD are trauma-focused CBT (such as prolonged exposure or cognitive therapy for PTSD) and Eye Movement Desensitization and Reprocessing (EMDR) [12, 80–82]. These therapies consist of several sessions involving detailed discussion of the trauma with a therapist—hence the term “trauma focused”. Recent studies indicate that a detailed focus on the trauma is not always necessary for PTSD symptoms to resolve, since while trauma focused CBT was superior a non-trauma-focused cognitive behavioural stress management therapy, the latter also led to a reduction of PTSD symptoms compared to waitlist control [83]. Trauma focused therapies are evolving from theory around reducing negative emotion such as fear via exposure to the fear in vivo or in imagination, as well as cognitive verbal reappraisal models [84]. An alternative way to update traumatic memories has been inspired by the application of theory on “memory (re)consolidation” to aversive memories [66]. Unlike exposure/extinction models, the latter forms of memory updating suggests only brief reminders to a memory are required and do not require strong emotion to be elicited.

Given the distress in recounting trauma that some patients experience taking part in trauma-focused psychotherapies, as well as the risks of secondary traumatisation to therapists repeatedly listening to details of trauma—there has been a wider interest in finding complementary forms of therapy to treat PTSD. For healthcare staff in particular, the work culture can discourage staff from talking openly about mental health problems [85]. Here we describe a few other emerging therapeutic approaches that are similar to ICTI in that they also seek to keep discussion of the trauma as brief as possible. A common element of some of these approaches with ICTI is that the focus is on mental imagery (imagination) and not just verbal cognition. A detailed review of all such techniques is beyond the scope of the current paper.

EMDR holds clear similarities with the idea of an imagery-competing cognitive task in terms of the working memory load theory underlying the eye movements component of EMDR [86]. It is thus worth noting (though beyond the scope of a formal review) that EMDR has for example been trialled for use with frontline emergency workers in response to the COVID-19 pandemic in the treatment of PTSD [87]. Briefer forms of EMDR are also being developed such as the “flash technique” which could offer a briefer low intensity form of treatment in a group format compared to multiple treatments session for EMDR [88]. The flash protocol involves choosing a disturbing memory, engaging focus on something positive, a distraction component (tapping) and ‘Flash’ (blinking rapidly). Studies to date indicate a reduction in distress associated with the targeted trauma memory (though impact on intrusive memory frequency is not known).

Intrusive images related to suicide (rather than past trauma) have been termed “flash-forwards” [89]. Since suicidal flashforwards are also mental imagery-based, they have the potential to be treated by imagery-competing task methods. A multicenter randomized clinical trial with depressed patients with suicidal mental imagery, found that a dual task add-on involving eye movements—in effect an imagery competing task—led to a reduction of severity and frequency of suicidal intrusions [90].

Imagery rescripting is a technique used in psychological therapies to approach the image and write and re-write the script. By imagining that the course of events is changed in a more desired direction, therapeutic effects have been found [91] and have shown good promising results over a number of studies[92, 93]. “Accelerated resolution therapy (ART)” [94] is described as a “predominately imaginative therapy” that addresses distressing events using imagery rescripting and metaphors. It aims to be brief and minimally distressing. Further, a technique also now drawing on ideas of reconsolidation-updating mechanisms is “Rewinding’ [95, 96]. As described by Danböck et al. (2024), individuals imagine their trauma on a movie screen from an observer perspective, with the scene starting right before the traumatic event took place (where the client was still safe) first, played forward, then, several times increasingly fast, backwards (up to ≈ 2–10 s depending on study protocol) to update the chronological sequence of the trauma memory [97].

Finally, we note that in the ICTI clinical trials that form the main focus of this paper the imagery-competing task used has been the computer game Tetris. Another hospital based study that tried a different task [98] did not find similar effects. We assume that it should be possible to use other tasks than Tetris with an ICTI protocol (i.e. alongside the other elements of mental rotation and a brief memory reminder, timing parameters etc.). However which imagery-competing tasks are optimal (or not do work) remains in need of further investigation. To this effect lab studies are useful [57, 99].

## Why does a new intervention approach such as ICTI matter in frontline healthcare settings?

If we already have talking therapies with an evidence base for helping to manage psychological trauma, why would it be important to develop interventions such as these? In essence, we know that frontline healthcare settings are busy spaces for both professionals and patients alike.

From a patient perspective, with any luck their encounter with frontline healthcare will be brief, however it may have the potential to leave deep scars. Further, the scale of need is vast, and there are simply too many people to help. For example, data from NHS digital suggest approximately 105,600 emergency caesarean sections a year in England [100]. If as many as 39% are at risk of developing PTSD, which might require a 8–12 session NICE recommended intervention, that would require approximately 550 psychological therapists working full time to tackle this need in England alone. Given the previous stated evidence base for the risk of developing more troubling psychological concerns, an easy access self-administered tool may reduce the costs to the NHS overall.

For staff, the 24–7 nature of healthcare including shift-work (and often unsocial hours), makes it harder to access conventional talking therapies in a timely manner. In addition, the exposure to work-related trauma is continuous, and as such a readily available “nip in the bud” approach would be beneficial. Also, there is little time for reflection and processing, so an active methodology for doing so could help. Staff may even come to the point in which the healthcare professional no longer recognises their experience as traumatic, or at least they do not feel able to name it as such. For instance, in a study in paediatric and neonatal intensive care, clinical staff were asked to rate their subjective levels of stress against objective measures of stress hormone in saliva samples. There was a significant discrepancy between how the staff rated their stress levels versus what their body indicated [101]. And so, staff may not be aware they need to access formalised pathways of psychological care, or not feel it applies to them. From a qualitative interview study about the experience of using competing task interventions staff (in the GAINS study) indicated feeling weak or stigmatised and not wanting others to know they were struggling as barriers in asking for support from mental health services and were positive about less stigmatising sources of help such as ICTI [102].

Positive experiences of using ICTI as a brief and flexible approach, as well as some challenges were also found in a qualitative interview study of staff in the EKUT-P study [103]. The guided session can be delivered by someone who is trained in ICTI used but does not necessarily need to be a qualified clinician, as shown in the EKUT-P and GAINS trials by the use of research assistants in the instructor role [7, 18, 79] akin to a digital navigator [104]. In addition, there may be a link between work performance and intrusive memories, whereby reducing IMs may help improve work functioning; intrusive memories can disrupt concentration [105] and adversely affect social and occupational functioning [16], which in turn may impact upon decision-making abilities and put a strain on working relationships with both colleagues and patients. Therefore, there is a functional performance and patient safety advantage for clinicians to reduce their experience of intrusive memories, and as swiftly as possible.

The imagery-competing task intervention (ICTI) approach holds advantages for patients and staff in frontline healthcare that also apply more generally post-trauma. It is brief (approximately 25 min per session, that is per each different intrusive memory) and can be used flexibly at any convenient time and place (for example, using a smartphone). It can be used preventatively as an early intervention post-trauma, as well as later as a treatment approach for established intrusive memories. It can be used for anyone who has the single symptom of intrusive memories and does not require a person to have a mental health disorder such as a diagnosis of PTSD, making it less stigmatising as an intervention approach. This also means that it can be accessed by self-identification of the symptom, rather than relying on referral by a clinician. It can be easily repeated (in a brief session) to address new/ongoing incidences of trauma or recurrence of intrusive memories, as well as used for single-event past trauma. Some people find discussing traumatic events in the way done in current evidence-based psychological therapies for PTSD difficult and distressing. The current intervention seeks to use a gentler approach by removing the need to discuss the trauma in detail, as the memory reminder cue uses just a few words and should be only a few seconds. Many people even report finding the intervention an enjoyable distraction rather than requiring a detailed discussion of distressing events.

It is too early to conclude that ICTI may be used beyond an intervention for IMs and as an stand-alone intervention for PTSD. The one trial with a 6 month follow up discussed earlier [79] found that ICTI led not only to a reduction in IMs but to other symptoms of PSTD, and that the benefits on wider PSTD symptoms were still present at 6 months. Some as yet unpublished data also examines whether ICTI has a domino effect on other symptoms of PTSD several months after the intervention. We also know from clinical practice that continued use of the intervention seems to have a possible preventative effect for continued exposure to work-related psychological trauma. Indeed, we envisage the utilisation of ICTI as a form of “mental health hygiene” for healthcare staff and others whose work environment lends itself to continued exposure to potential psychological trauma.

Lastly, but perhaps most importantly, in countries where psychological healthcare is less available, a simple, easy to access, scalable intervention would be prudent, and although this intervention still requires some mental health professional support alongside, it shows promise of scalability.

## Conclusion

Experience of psychological trauma spans all people, with particular experience in healthcare populations and the healthcare staff who treat them. However most psychological interventions to date require ongoing treatment within a psychotherapy setting and are designed to treat PTSD as a syndrome. This paper summarised the thinking behind targeting IMs as a transdiagnostic symptom, working beyond PTSD as a diagnosis. It outlined the learning from cognitive science that interference in the (re)consolidation of visual memories by utilisation of procedure including imagery competing tasks has the potential to act as an intervention for IMs. Over the last years, the work from our group and others has been to optimise and trial an imagery-competing task intervention that includes the widely accessible computer game Tetris®. The intervention in these first trials has shown efficacy in healthcare patients and healthcare staff. It has shown efficacy in both early intervention after trauma and with longer term experience of established IMs. It is both scalable and acceptable to participants and holds the benefit of being able to use it flexibly in different places (in a hospital bed, in the staff room, on a commute or at home). The intervention so far has been tested as a standalone approach, but future studies could investigate approaches in combination with other mental health treatments. In addition, further investigation into the brain mechanisms of the current intervention is warranted, as well as how to optimise reach of this scalable intervention approach.

## Data Availability

No datasets were generated or analysed during the current study.

## Footnotes

1 The sample was across 24 countries from a series of general population studies carried out throughout the world from 2001 to 2012. Including USA and Europe in there as well as lower income countries but not including UK data.
